# ‘*Nedoceratops*’: An Example of a Transitional Morphology

**DOI:** 10.1371/journal.pone.0028705

**Published:** 2011-12-14

**Authors:** John B. Scannella, John R. Horner

**Affiliations:** Museum of the Rockies and Department of Earth Sciences, Montana State University, Bozeman, Montana, United States of America; College of the Holy Cross, United States of America

## Abstract

**Background:**

The holotype and only specimen of the chasmosaurine ceratopsid dinosaur ‘*Nedoceratops hatcheri*’ has been the source of considerable taxonomic debate since its initial description. At times it has been referred to its own genus while at others it has been considered synonymous with the contemporaneous chasmosaurine *Triceratops*. Most recently, the debate has focused on whether the specimen represents an intermediate ontogenetic stage between typical young adult *Triceratops* and the proposed mature morphology, which was previously considered to represent a distinct genus, ‘*Torosaurus*’.

**Methodology/Principal Findings:**

The only specimen of ‘*Nedoceratops hatcheri*’ was examined and the proposed diagnostic features of this taxon were compared with other chasmosaurine ceratopsids. Every suggested autapomorphy of ‘*Nedoceratops*’ is found in specimens of *Triceratops*. In this study, *Triceratops* includes the adult ‘*Torosaurus*’ morphology. The small parietal fenestra and elongate squamosals of *Nedoceratops* are consistent with a transition from a short, solid parietal-squamosal frill to an expanded, fenestrated condition. Objections to this hypothesis regarding the number of epiossifications of the frill and alternations of bone surface texture were explored through a combination of comparative osteology and osteohistology. The synonymy of the three taxa was further supported by these investigations.

**Conclusions/Significance:**

The *Triceratops*, ‘*Torosaurus*’, and ‘*Nedoceratops*’ morphologies represent ontogenetic variation within a single genus of chasmosaurine: *Triceratops*. This study highlights how interpretations of dinosaur paleobiology, biodiversity, and systematics may be affected by ascribing ontogenetic and other intraspecific variation a taxonomic significance.

## Introduction

For many years after the description of the first species of *Triceratops* (*T. horridus*) [1], nearly every variation in cranial morphology between specimens was considered sufficient grounds to erect new species. By 1949, as many as 16 species of this genus had been named [2], [3], [4]. After his initial description of *Triceratops*, O.C. Marsh also named two species of a new genus of latest Cretaceous ceratopsid (‘*Torosaurus*’), which were found in the same geological formation and geographic area as the *Triceratops* specimens [5]. ‘*Torosaurus*’ differed from *Triceratops* in having an expanded, fenestrated parietal and elongate squamosals. The resultant high number of apparently coeval taxa had major implications for interpretations of dinosaur paleoecology and end-Cretaceous taxonomic diversity [2].

Ostrom and Wellnhofer [2], [6] called attention to the apparent high diversity of *Triceratops* and proposed an alternative hypothesis: that the variation used to erect all of these taxa was instead simply intraspecific variation within *T. horridus*. They noted similar levels of variation in populations of extant horned mammals. This idea was largely supported by the more recent work of Forster [4], who found morphometric evidence for only two species of *Triceratops* (*T. horridus* and *T. prorsus*). Ostrom and Wellnhofer further suggested that ‘*Torosaurus*’ may actually represent sexual dimorphism within *Triceratops* [6]. We recently presented evidence that ‘*Torosaurus*’ instead represents the mature morphology of *Triceratops* [7]. As *Triceratops* matured, its skull underwent a series of radical transformations: the postorbital horn cores changed orientation, the epiossifications (epoccipitals) bordering the parietal-squamosal frill changed shape, and the frill itself expanded and became fenestrated [7], [8], [9], [10]. The end-product of this transformation was the morphology previously considered to represent a distinct genus: ‘*Torosaurus*’. Consideration of potential sources of intraspecific variation, including ontogenetic change, has reduced 18 latest Cretaceous ceratopsid taxa to two (*Triceratops horridus* and *Triceratops prorsus*). This produces a dramatically different view of horned dinosaur systematics.

**Figure 1:**
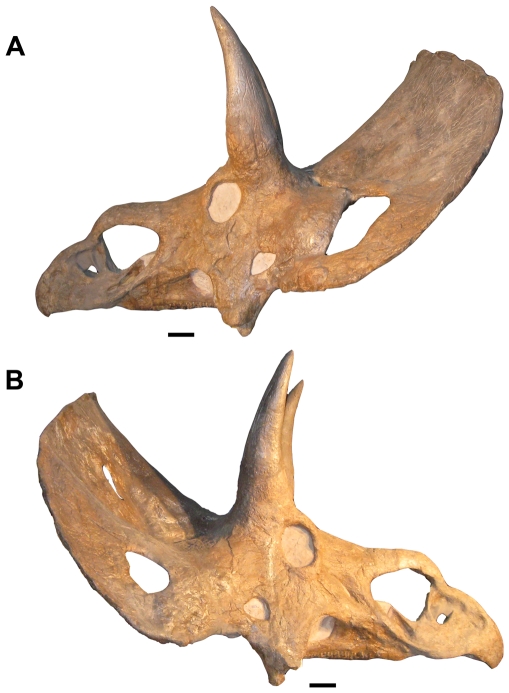
USNM 2412, the holotype and only specimen of ‘*Nedoceratops hatcheri*’. **A.** Left lateral view. **B.** Right lateral view. Scale bars equal 10 cm.

One of the 18 proposed taxa, represented by a single skull (USNM 2412; Figure 1), has had a particularly complex taxonomic history. It has at various times been considered a distinct genus (‘*Diceratops*’; [4], [11]), a species of *Triceratops* (‘*Triceratops hatcheri*’; [12]), or variation within *Triceratops horridus* [2], [13]. The genus name ‘*Diceratops*’ was recently found to have been preoccupied and two new names were proposed: ‘*Diceratus*’ [14] and ‘*Nedoceratops*’ [15]. ‘*Nedoceratops*’, being published first, has priority [16]. The prefix ‘nedo’ is Russian in origin and means ‘insufficiency’ [15] – thus, ‘*Nedoceratops*’ roughly translates to ‘insufficient horned face.’

USNM 2412 has been considered unusual in that unlike the holotypes of *Triceratops* it expresses a small parietal fenestra and fenestrated squamosals [11]. Dodson [3] noted that the slightly elongate squamosals of USNM 2412 bear a closer resemblance to those of ‘*Torosaurus*’ than to *Triceratops*. The specimen also has a greatly reduced or perhaps absent epinasal [4]. We have previously noted that several of the unusual features of USNM 2412 appear to be intermediate between those seen in *Triceratops* and ‘*Torosaurus*’, and proposed that the specimen represents a *Triceratops* which was beginning to develop the expanded, fenestrated frill characteristic of the mature morphology, ‘*Torosaurus*’ [7]. Recently, Farke [17] redescribed USNM 2412 and considers ‘*Nedoceratops hatcheri’* a valid taxon, stating that its unusual features “may be explained as a whole suite of abnormalities in a single aberrant individual of *Triceratops*, or a suite of autapomorphies characterizing a taxon distinct from *Triceratops* (pg. 6).” Farke favors the latter hypothesis and rejects the hypothesis that ‘*Torosaurus*’ represents a mature *Triceratops* (the Ontogenetic Trajectory Hypothesis [OTH] as he terms it).

The degree to which some proposed dinosaur taxa may or may not actually represent variation (either ontogenetic or individual) within established taxa is debated (e.g., [7], [17], [18], [19]). Ontogenetically transitional morphologies in the dinosaur fossil record have the potential to dramatically affect interpretations of taxonomy and systematics (e.g., [7], [20], [21], [22], [23]). The taxonomic status of USNM 2412 (as well as ‘*Torosaurus*’ and *Triceratops*) has significant implications for trends in dinosaur diversity preceding the K/Pg boundary.

Here we will demonstrate that every feature of USNM 2412 proposed to be diagnostic of a distinct genus is found within *Triceratops* and thus USNM 2412 more likely reflects variation within *Triceratops*. We also provide further evidence for the synonymy of *Triceratops* and ‘*Torosaurus*’ (by ‘*Torosaurus*,’ we are referring to ‘*Torosaurus latus*’, not ‘*Torosaurus*’ *utahensis* [7]). Finally, we discuss the effect of intraspecific variation on interpretations of dinosaur paleobiology and systematics.

**Institutional abbreviations.** AMNH, American Museum of Natural History, New York, New York, USA; ANSP, Academy of Natural Sciences of Philadelphia, Pennsylvania, USA; CM, Carnegie Museum, Pittsburgh, Pennsylvania, USA; MOR, Museum of the Rockies, Bozeman, Montana, USA; MPM, Milwaukee Public Museum, Milwaukee, Wisconsin, USA; RTMP, Royal Tyrrell Museum, Drumheller, Alberta, CA; UCMP, University of California Museum of Paleontology, Berkeley, California, USA; USNM, National Museum of Natural History, Washington D.C., USA; YPM, Yale Peabody Museum, New Haven, Connecticut, USA.

## Results

### Reassessment of USNM 2412

Farke [17] diagnoses ‘*Nedoceratops hatcheri*’ as follows:

“Chasmosaurine ceratopsid with the following autapomorphies: nasal horncore nearly completely undifferentiated from the nasal bone; greater portion of procurved postorbital horncores forms 90 degree angle with tooth row; and parietal fenestrae extremely small (occupying less than five percent of the total surface area of the parietal). *Nedoceratops hatcheri* is distinguished from *Triceratops* spp. in the position of the ventral extremity of the squamosal well above the alveolar process of the maxilla, and in the presence of parietal fenestrae, which are lacking in *Triceratops* species. *Nedoceratops hatcheri* is distinguished from *Torosaurus latus* in squamosal shape (particularly the reduced jugal notch and lack of a thickened medial margin in *N. hatcheri*), and that *N. hatcheri* has extremely reduced parietal fenestrae and a low number of episquamosals relative to *T. latus*. (pg. 4)”

#### Nasal and Nasal Horn

**Figure 2:**
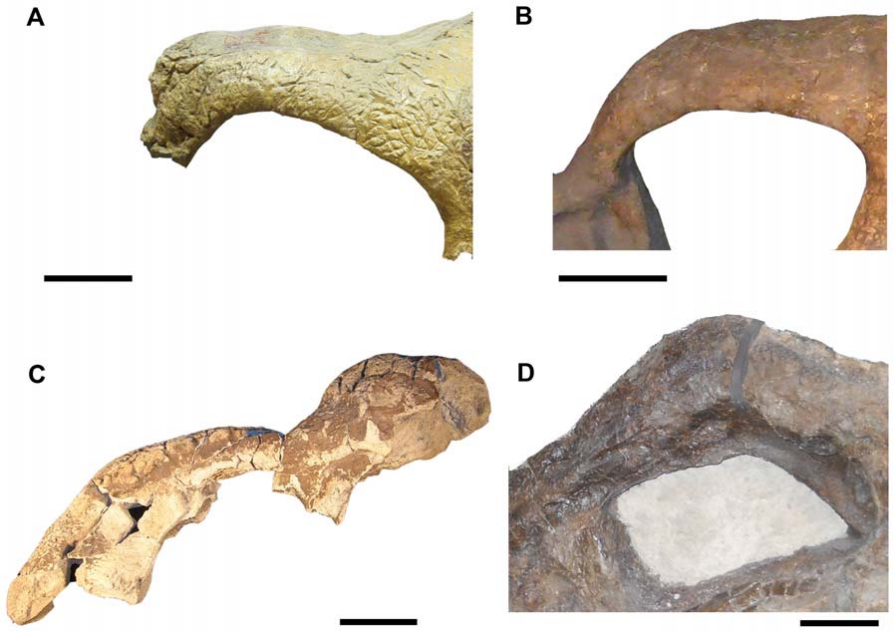
Nasal horn variation in *Triceratops*. **A.** USNM 4720, originally named the holotype of *Triceratops* ‘*obtusus*.’ This specimen preserves a very low, blunt nasal horn. **B.** USNM 2412, the holotype of ‘*Nedoceratops hatcheri*.’ The nasal horn of this specimen (if present – see discussion) is a low, smooth boss. **C.** UCMP 128561, originally named the holotype of ‘*Ugrosaurus olsoni*.’ The nasal horn of this specimen is a low rugose boss. **D.** MOR 981 (previously ‘*Torosaurus*’). This specimen bears a low boss which is undifferentiated from the nasals. Scale bars equal 10 cm.

The nasal horn of USNM 2412 is indeed ‘nearly completely undifferentiated from the nasal bone’, however similar poorly defined nasal horn morphologies are seen in several specimens of *Triceratops* (UCMP 128561, USNM 1208, USNM 4720, MOR 981, MOR 1122; Figure 2). The nasal horn of MOR 981 (Figure 2D) is particularly unpronounced, yet not quite to the degree observed in USNM 2412. It has been suggested that the epinasal of USNM 2412 was lost either taphonomically or due to pathology [2], [4], [9]. Areas of the anterior nasals are obscured by reconstruction; however there is no clear evidence of breakage indicating a missing epinasal. Farke [17] suggests that the lack of an open epinasal suture makes it improbable that the epinasal was lost due to trauma in life. However the epinasal is known not to fuse to the underlying nasals until fairly late in ontogeny in some specimens of *Triceratops* [9]. If the epinasal was lost in life and the nasal sutures proceeded to close, there would be no reason to expect an open epinasal suture. For these reasons, the nasal horn morphology of USNM 2412, taken on its own, presents insufficient grounds to distinguish this specimen from *Triceratops*.

#### Postorbital Horn Cores

The left postorbital horn core is largely reconstructed, yet preserves the base which is slightly anteriorly inclined (Figure 1). The right postorbital horn core is more complete, and is fairly erect (varying from the morphology expressed on the left side of the skull) yet procurved. The orientation of the postorbital horn cores undergoes a radical change throughout ontogeny in *Triceratops* [8]. In the smallest (‘baby’) specimens, the postorbital horns are erect, in juveniles they begin to curve posteriorly and then as ontogeny progresses they become procurved. Procurving of the postorbital horn cores indicate that USNM 2412 was a fairly mature individual (see Discussion). Given that the postorbital horn cores transformed so dramatically throughout ontogeny, variation in orientation between specimens is expected. Intraspecific variation in the orientation of the base of the postorbital horn core has been demonstrated in several taxa of chasmosaurine ceratopsids [24]. Thus, the orientation of horn cores cannot be used to distinguish USNM 2412 from *Triceratops*.

#### Parietal Fenestrae

**Figure 3:**
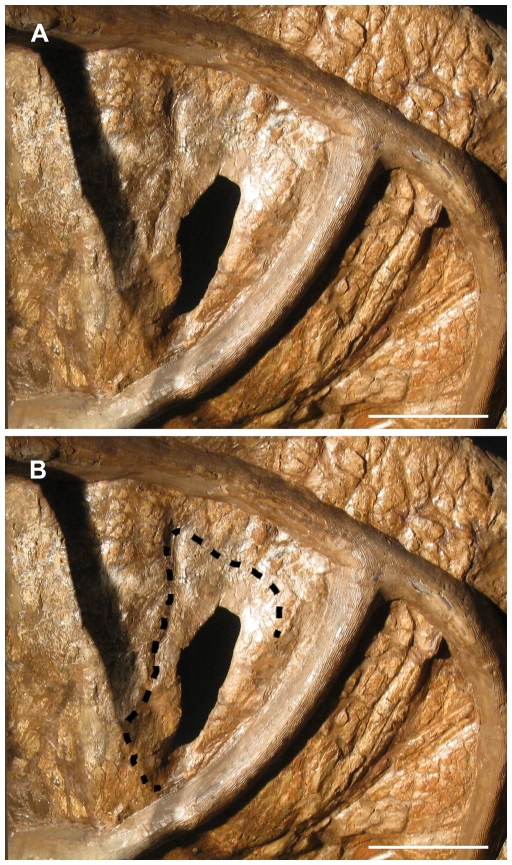
Ventral view of the right half of the parietal of USNM 2412. **A.** When viewed with offset lighting, the rim of a shallow depression surrounding the small fenestra is apparent. **B.** Extent of the depression is outlined. The area within the outline is markedly thinner than the remainder of the parietal. The extent of the depression is partially obscured by the framework which supports the skull. Scale bars equal 10 cm.

**Figure 4:**
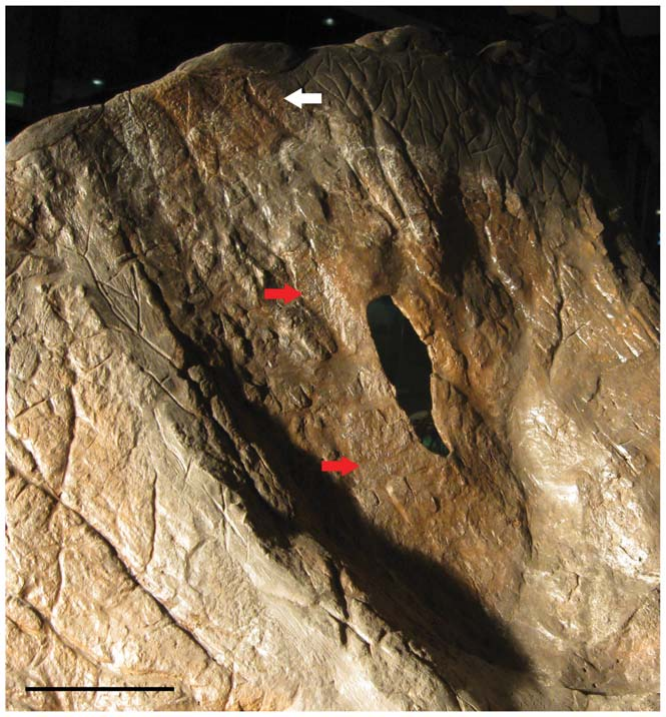
Dorsal view of the parietal fenestra of USNM 2412. Although much of the parietal is obscured by reconstruction, a transition in surface texture from the posterior margin (white arrow) to the area immediately adjacent to and surrounding the fenestra (red arrows) is apparent. Scale bar equals 10 cm.

Arguably the most intriguing feature of USNM 2412 is the presence of a small parietal fenestra on the right side of the frill (the left half of the parietal is largely unpreserved). Farke [17] notes no ventral depression surrounding the parietal fenestra of USNM 2412, though acknowledges that the parietal is extremely thin in this region. There is, in fact, a subtle ventral depression around the parietal fenestra of USNM 2412 which conforms to the shape of the fenestra (though it is slightly obscured by the metal framework supporting the skull and not immediately obvious upon observation; Figure 3). Also, there is a transition in surface texture surrounding the fenestra (Figure 4): the area immediately adjacent to the fenestra is somewhat less rugose than the rest of the parietal (though much of the surface texture is obscured by reconstruction). Parietal fenestrae are unknown in *Triceratops* (exclusive of ‘*Torosaurus*’ specimens) but are found in specimens referred to ‘*Torosaurus*’. As we previously noted [7], if *Triceratops* matured into the morphology previously considered diagnostic of ‘*Torosaurus*’, at some point the parietal fenestrae would open. It is predicted that the fenestrae would start off small and expand in size, as is seen in centrosaurine ontogeny [19], [25], [26]. Thus, we interpret the small parietal fenestra of USNM 2412 as a product of the parietal thinning which we previously demonstrated [7] and not diagnostic of a distinct taxon (see Discussion).

#### Squamosal morphology

**Figure 5:**
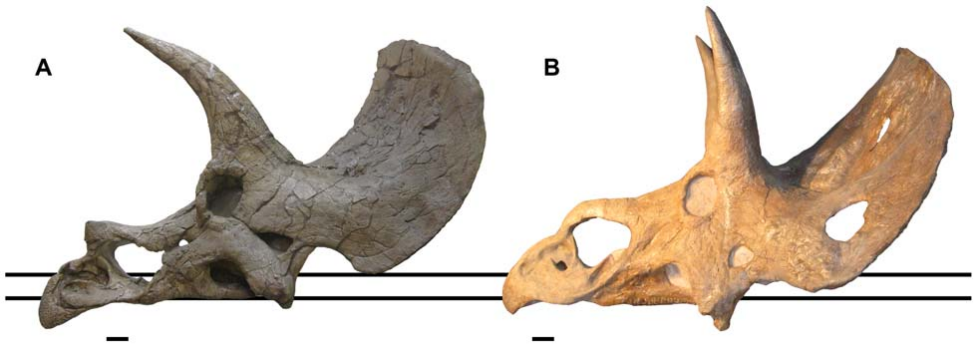
Lateral views of USNM 1201 and USNM 2142. **A.** Left lateral view of USNM 1201, originally named the holotype of *Triceratops* ‘*elatus*.’ Note that the ventral extremity of the squamosal (denoted by upper horizontal line) is positioned well above the alveolar process of the maxilla (denoted by lower horizontal line). **B.** USNM 2412, right lateral view (reversed for direct comparison with USNM 1201 which only preserves the left side of the skull; the right squamosal of USNM 2412 is more elevated than the left). The alveolar process of the maxilla is positioned on the lower horizontal line, allowing for a direct comparison with USNM 1201. Note that the squamosal is not elevated to the extent found in USNM 1201. The position of the ventral extremity above the alveolar process of the maxilla can thus not be used to distinguish ‘*Nedoceratops hatcheri’* from *Triceratops*. Scale bars equal 10 cm.

The ventral extremity of the squamosal of USNM 2412 is positioned above the alveolar process of the maxilla. A similar configuration is seen in several specimens of *Triceratops* (e.g., UCMP 113697; USNM 1201) and is thus not diagnostic of a distinct taxon (Figure 5). Similarly, there is considerable variation in the size of the jugal notch formed by the squamosal in *Triceratops* specimens (see Figure 3 in [7]). Indeed, there is variation in the size of the jugal notch between the left and right side of USNM 2412 (Figure 1). This is likely due to pathology [27]. The lack of a thickened median margin of the squamosal (or ‘squamosal bar’ [28]) exhibited by USNM 2412 is expected until late in ontogeny in *Triceratops* [7]. The squamosals bear asymmetrical fenestrae which, as Farke [17] notes, are not reliable for taxonomic purposes. The squamosal morphology of USNM 2412 is here interpreted as representing an intermediate between the short, broad condition typical of immature *Triceratops* and the elongate morphology with a thickened median margin which is found in mature specimens (previously assigned to ‘*Torosaurus*’).

#### Episquamosal number

The episquamosal count of USNM 2412 has been estimated as five, with the rostral episquamosals indiscernible due to tight fusion [17]. Alternatively, we suggest that the rostral episquamosals are not preserved in this specimen. Episquamosals are commonly lost taphonomically [9]. The number of episquamosals preserved on USNM 2412 cannot be used to distinguish it from *Triceratops* (as noted by Farke [17]) and as such does not support the hypothesis that this specimen represents a distinct genus. The epiparietals of USNM 2412 are reconstructions [17].

## Discussion

### Taxonomic Status of ‘*Nedoceratops hatcheri*’

All of the features and conditions used to diagnose ‘*Nedoceratops*’ are found in *Triceratops*. The nasal horn, if present, is greatly reduced but given the variation in nasal horn morphology in *Triceratops* ([2], [17], [29],[30] contra [31]) and the fact that other specimens possess nasal horns which are not much larger than that expressed by USNM 2412, we interpret this as variation within *Triceratops*. Alternatively, as noted above, the epinasal may have been lost in vivo – in which case this feature would not be useful for taxonomic diagnoses. The squamosal morphology (particularly that of the right side of the skull, the left squamosal apparently being pathologic [27]) does not fall outside the range of variation found in *Triceratops* (see [7]). The horn cores are procurved yet the right horn core is more erect than is typical in *Triceratops*. A similarly erect horn core is found in the holotype of *Eotriceratops xerinsularis* (RTMP 2002.57.7; [32]). We interpret this variation in postorbital horn core orientation as a result of the morphological change which these elements undergo throughout ontogeny [8]. Variation between the orientations of the left and right postorbital horn cores in USNM 2412 further suggests that this feature is of limited taxonomic utility. Indeed, even if ‘*Torosaurus*’ is not synonymous with *Triceratops*, the only feature which unambiguously distinguishes ‘*Nedoceratops*’ from *Triceratops* is the small parietal fenestra. However, we think it is more likely that had the animal not died when it did that this fenestra would have continued to develop and the frill would have continued to expand, resulting in a morphology indistinguishable from mature *Triceratops* (‘*Torosaurus*’).

### Taxonomic Status of ‘*Torosaurus latus*’

Radical ontogenetic changes in cranial morphology have been noted in several dinosaur taxa (e.g., [19], [21], [22], [33], [34]). *Triceratops* underwent a dramatic cranial transformation throughout ontogeny - the postorbital horn cores completely changed orientation and prominent triangular epiossifications of the cranial frill increased in size in juveniles and subadults and then became resorbed and flattened in more mature individuals [8], [9]. We have proposed that the cranial transformation of *Triceratops* included an expansion of the parietal-squamosal cranial frill, ultimately leading to the thin, fenestrated condition previously considered diagnostic of *‘Torosaurus latus*’ [7]. The proposed synonymy of *Triceratops* and ‘*Torosaurus*’ (the ‘OTH’ [17]) has been challenged based on observations about the number and position of epiossifications on the cranial frill and cranial surface texture [17].

#### Variation in epiossification number and position

As Farke notes, *Triceratops* specimens typically express five to seven episquamosals, whereas ‘*Torosaurus*’ specimens have seven [7], [17], [35]. Farke states that “even squamosals from “baby” and juvenile *Triceratops* have between five and seven scallops for attachment of episquamosals . . . corresponding precisely to the number found in most adult-sized individuals (pg. 7).” This also corresponds to the number of episquamosals found in ‘*Torosaurus*’ specimens (e.g., MOR 1122, MPM VP6841). Thus, variation in episquamosal number does not falsify the OTH. The alternative is to ascribe different species names to specimens with five, six, or seven episquamosals. As the number of episquamosals has been found to vary between the left and right sides of individuals [7] and these elements are easily lost taphonomically [9], assigning species names based solely on this criteria would very likely be erroneous.

*Triceratops* has been diagnosed as possessing both a midline epiparietal and epiossifications which span the parietal-squamosal contacts [4]. Specimens typically possess five or six epiparietals, not including the epiossifications which cross the parietal-squamosal contacts (e.g., [4], [7]). ‘*Torosaurus*’ specimens, on the other hand, have been found to express evidence for between 10 (MOR 981) and 12 (MOR 1122) epiparietals [28]. These specimens do not express a midline epiparietal or epiossifications spanning the parietal-squamosal contacts. Thus, if ‘*Torosaurus*’ represents the mature morphology of *Triceratops* it means that a significant reconfiguration of epiparietals occurred throughout ontogeny.

The most complete ‘*Torosaurus*’ parietal is that of MOR 1122, a specimen which clearly expresses 12 epiparietals. Direct comparisons of this specimen to (non-‘*Torosaurus*’) *Triceratops* suggest an increase of at least six epiparietals throughout ontogeny. Is such a transformation feasible? Farke [17] notes that an addition of epiossifications is apparently not found in ontogenetic series of centrosaurines (though he acknowledges variation by as many as two epiparietals and one episquamosal between juveniles and adults, a difference which he considers individual variation). We question whether direct comparisons of frill epiossifications of chasmosaurines and centrosaurines are, in this regard, appropriate. In so far as chasmosaurine ceratopsids, Forster et al. [36] and Godfrey and Holmes [37] noted an apparent increase in episquamosals throughout ontogeny in *Agujaceratops mariscalensis* and *Chasmosaurus* spp. (respectively). Thus the suggestion of an addition of epiossifications throughout ontogeny in chasmosaurines is by no means unprecedented. These two examples may alternatively be interpreted as individual variation [17].

**Figure 6:**
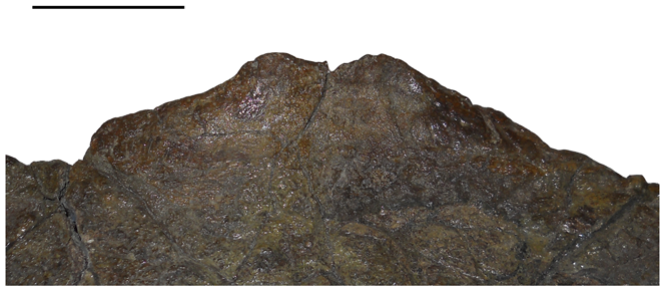
Episquamosal of MOR 2975. The presence of two peaks is suggestive of midline erosion. Scale bar equals 5 cm.

*Triceratops* frill epiossifications undergo dramatic changes in morphology throughout ontogeny [8], [9]. They expand, elongate, and eventually flatten. Forster [35] noted that in one specimen of *Triceratops* (CM 1221) these elements ‘fuse into a continuous epoccipital rim (pg. 252).’ A specimen at the MOR (MOR 2975) exhibits an episquamosal with two peaks, which is suggestive of erosion of the midline of the element and eventual division had elongation continued (Figure 6). If the six epiparietals of a (non-‘*Torosaurus*’) *Triceratops* were each to elongate and divide throughout ontogeny, it would produce 12 epiparietals. Osteohistological studies have already established that dinosaur cranial adornments were capable of dramatic transformations throughout ontogeny (likely due to metaplastic transformation [10], [22]); continued erosion of the epiossifications to eventually divide the elements is thus not an unfeasible mechanism for the apparent addition of epiossificatons throughout ontogeny which has been previously hypothesized [7], [12], [36], [37].

Another factor which must be considered in concert with ontogenetic transformation is stratigraphic variation [38]. MOR 1122, which expresses 12 epiparietals, was collected from the bottom of the Hell Creek Formation in eastern Montana. No other *Triceratops* specimens have been reported from this low in the formation. MOR 981, originally referred to ‘*Torosaurus*’ [28], was collected from slightly higher in the formation. As Farke [28] noted, MOR 981 exhibits evidence of only ten epiparietals. MPM VP6841, a large specimen previously referred to ‘*Torosaurus*’ [39], was collected from significantly higher in the formation [40]. It does not exhibit a complete set of epiparietals, however the one most completely preserved epiparietal is approximately 35 cm in length. Given the total width of this specimen's parietal (204 cm), the maximum number of epiparietals it could have accommodated – assuming no spaces between each epiparietal – is approximately six, a number comparable to that found in specimens of (non-‘*Torosaurus*’) *Triceratops* [9]. There is likely a stratigraphic component to epiparietal count; the count suggested by MPM VP6841 is in agreement with *Triceratops*.

**Figure 7:**
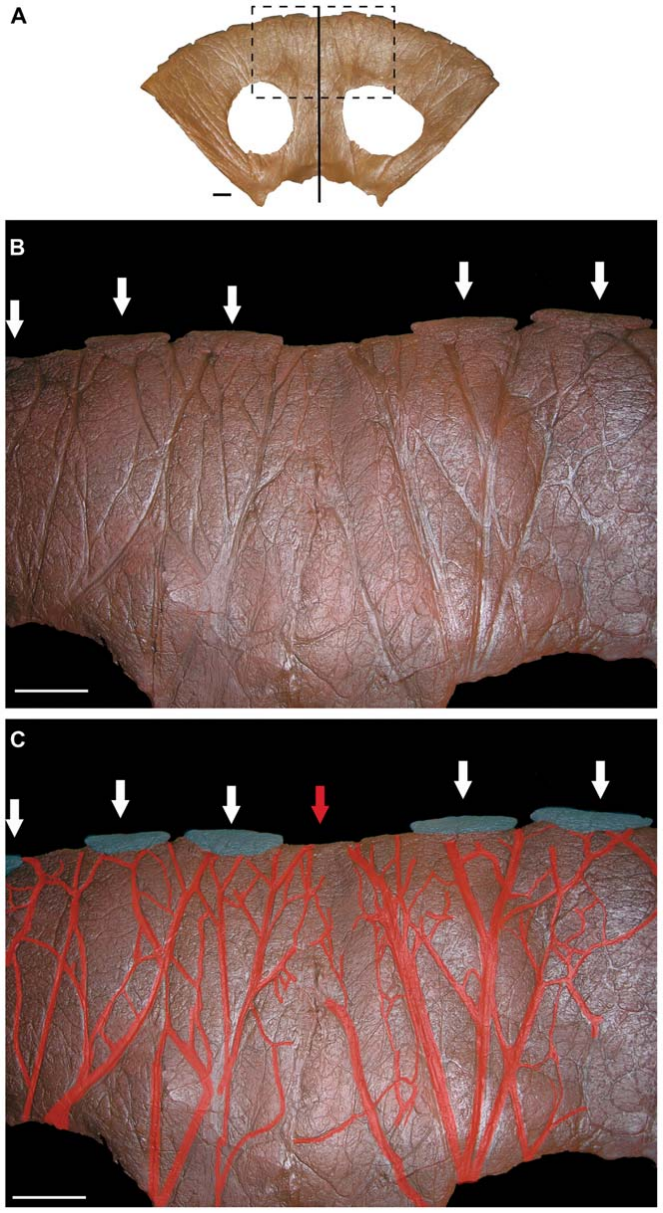
Ventral view of the parietal of MOR 1122. **A.** The entire parietal with midline denoted by vertical line. Dashed rectangle indicates area of interest in B and C. **B.** Impressed vascular traces are found over the entire ventral surface of the parietal. Epiparietals are indicated by arrows. MOR 1122 does not appear to possess an epiparietal over the midline of the parietal. **C.** Major vascular traces are highlighted in red. Note that the most prominent vascular traces lead to the epiparietals (highlighted in blue). Two large vascular traces lead to the midline of the parietal (denoted by red arrow), suggesting that an epiparietal occupied this position but was lost taphonomically. Scale bars equal 10 cm.

Furthermore, the presence of an epiossification spanning the parietal-squamosal contact, though previously unreported, is clearly present in a specimen of ‘*Torosaurus*’ (ANSP 15192; see Figure 8 in [7]). There is also evidence suggesting a midline epiparietal was present on MOR 1122, though unpreserved: vascular traces on the ventral surface of the parietal appear to lead directly from the anterior region of the parietal to each epiparietal (Figure 7). Pronounced vascular traces clearly terminate at the midline of the parietal, suggesting that blood was supplied to a midline epiparietal. If so, this further supports the synonymy of these taxa.

#### The parietal of USNM 2412 as intermediate between *Triceratops* and ‘*Torosaurus*’

If the parietal-squamosal frill of *Triceratops* eventually adopted the morphology previously considered diagnostic of ‘*Torosaurus*’ through ontogeny, the discovery of intermediate morphologies would be expected. The existence of such intermediate specimens has been previously documented: throughout ontogeny the squamosals elongated and the parietal developed thin regions in the same areas where specimens of ‘*Torosaurus*’ have fenestrae [7], [10]. USNM 2412 represents an important specimen in that it exhibits a small parietal fenestra in the same region of the parietal where thin areas (histologically revealed to have been actively becoming thinner at the time of death [7], [10]) are found in *Triceratops*. We consider these regions ‘incipient fenestrae.’ Farke suggests that these thin areas were instead areas for muscle attachment [17], [41]. As noted above, both a ventral depression and transition in surface texture are found around the parietal fenestra of USNM 2412. A transition in surface texture has been noted around the developing fenestrae of AMNH 5116 (*Triceratops* [7]) and in centrosaurines [19], [26]. AMNH 5116 expresses a striated surface texture over much of the parietal, and a ‘pebbly’ surface texture in the regions which we hypothesize represent developing fenestrae. USNM 2412 differs slightly from this, in that no striated texture appears to be present on the parietal (though, as noted, much of the parietal's surface is obscured by reconstruction). Instead there is a transition from rugose (‘adult’) surface texture to the less rugose texture around the fenestra.

**Figure 8:**
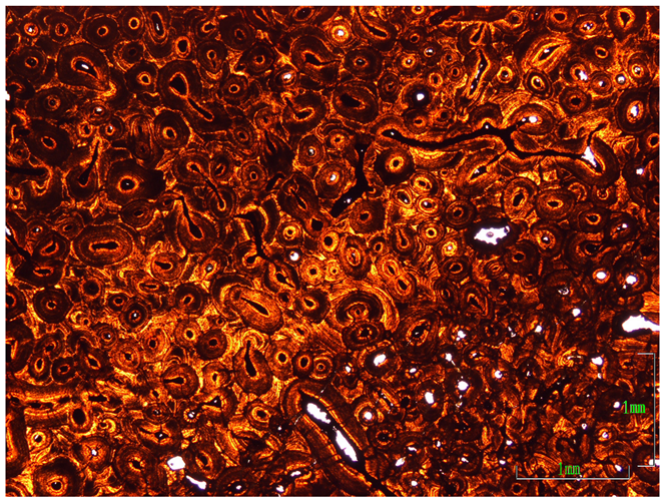
Osteohistology of the postorbital horn core of MOR 981. The dense, multigenerational ‘Haversian’ tissue is indicative of a mature individual.

The presence of rugose surface texture on the parietal of USNM 2412 has been noted as evidence of an ‘old-adult’ ontogenetic status for the specimen [17]. Centrosaurine frills appear to have passed through three sequential ontogenetic stages: 1) ‘long-grained’ surface texture in juveniles; 2) mottled surface texture; 3) smooth/rugose ‘adult’ texture [19], [26], [42]. Transformation of ceratopsid frill surface texture which does not follow this sequence, or reverts back and forth between these surface textures, has not previously been described (however Ryan and Russell [43] note a ‘modified long-grain bone texture’ on cranial elements of large *Centrosaurus brinkmani*). It is important to emphasize that surface textures represent expressions of the histological growth and remodeling processes which were occurring at the time of the animal's death [42]. ‘Long-grained’ or striated texture, for example, is associated with rapid bone expansion [19], [26], [42], [44]. The parietal of MOR 981 (‘*Torosaurus*’) expresses a striated texture over much of its surface [7]. Histological examination of this parietal reveals that it was expanding at the time of death [10]. Based on this observation, it might be predicted that MOR 981 was less mature – perhaps significantly so – than MOR 1122, a ‘*Torosaurus*’ specimen which expresses histological evidence of extreme maturity and exhibits rugose surface texture on its parietal [7]. However, examination of the osteohistology of the postorbital horn core of MOR 981 reveals extremely dense, multigenerational ‘Haversian’ tissue indicative of maturity equivalent to that seen in MOR 1122, and greater than that expressed in (non-‘*Torosaurus*’) *Triceratops* (Figure 8). The presence of surface striations indicative of rapid expansion of the parietal in very mature specimens of *Triceratops* (‘*Torosaurus*’) supports the hypothesis that the short, thickened frill of a young adult *Triceratops* expanded and thinned late in ontogeny.

Farke notes that surface textures associated with bone resorption, such as those observed on the parietals of *Triceratops* and other ceratopsids, are not unambiguously associated with the formation of fenestrae [17]. This is true, as resorption occurs in all bones – it is a general feature of growth and remodeling [44], thus the presence of mottled surface textures in areas of ceratopsid skulls that do not form fenestrae (such as the parietal midline [26]) does not imply the formation of fenestrae but it does indicate that bone resorption was occurring in those areas at the time of death. In areas where fenestrae do form, histological evidence of resorption would be expected and has been demonstrated [7], [10]. The histological evidence suggests that reversion from a rugose (‘adult’) surface texture to a striated texture occurred in *Triceratops*. Therefore, the presence of rugose surficial texture on the parietal of USNM 2412 does not indicate that it had stopped growing, or that its parietal would not have undergone further changes had it not died when it did.

The suggestion that the thin regions of the parietal in *Triceratops* were areas for muscle attachment [17], [41] would be supported by the presence of abundant extrinsic fibers in these areas [45]. Examination of the microstructure of these areas in *Triceratops* reveals no evidence of extrinsic fibers and thus suggests that these were not regions of indirect muscle attachment. However, it is possible there was a direct attachment to the periosteum [45]. This is a somewhat more difficult hypothesis to test histologically with fossil material. Significantly, if the cranial ornamentation of marginocephalians developed through metaplastic transformation, as has been suggested [10], [22], it would indicate the absence of a periosteum at some point in ontogeny and thus direct muscle attachment would be unlikely. Regardless of whether or not there were muscle attachments in these regions at some point in ontogeny, the fact that USNM 2412 exhibits a fenestra within this area of the parietal confirms that resorption leading to eventual fenestration occurred here. We interpret USNM 2412 as a transitional morphology between the solid parietal of immature *Triceratops* and the fenestrated condition of mature individuals (‘*Torosaurus*’).

#### Juvenile ‘*Torosaurus latus*’

Until a clearly juvenile ‘*Torosaurus*’ is recovered - with backwards curving postorbital horn cores, delta-shaped frill epiossifications, elongate squamosals, and a fenestrated parietal – it appears more likely that either: a) juvenile ‘*Torosaurus*’ were largely indistinguishable from *Triceratops* and differences in morphology between these two taxa only became apparent later in ontogeny, or b) ‘*Torosaurus*’ and *Triceratops* are synonymous. We favor the latter hypothesis for reasons previously discussed in detail [7]. An extensive, unbiased survey of the fossils of the Hell Creek Formation resulting in the collection of numerous rare ontogenetic stages of dinosaur taxa failed to recover evidence of juvenile ‘*Torosaurus*’ clearly distinguishable from *Triceratops* [7], [23], [46].

Farke [17] suggests that YPM 1831 (initially described as the holotype of ‘*Torosaurus gladius*’ [5]) may represent a subadult ‘*Torosaurus*.’ He notes that the frill of this specimen exhibits a smooth surface texture, no epiossifications are readily visible, and that several cranial elements are unfused. The frill of YPM 1831 exhibits a striated surface texture similar to that observed in MOR 981. As noted previously [7], this surface texture is found in several specimens expressing indicators of ontogenetic maturity. As discussed above, MOR 981 itself exhibits extremely mature bone histology, thus the surface texture of the frill of YPM 1831 does not indicate subadult status. The postorbital horn cores of YPM 1831 are procurved, which is further indicative of ontogenetic maturity. The fact that epiossifications were not fused to the frill margin does not indicate a subadult status as many young adult/adult *Triceratops* do not retain all of their epiossifications (e.g., AMNH 5116, MOR 981, MOR 1122, MOR 2702, MPM VP6841, YPM 1830). As noted previously, epiossifications appear to have been easily lost taphonomically. There is considerable variation in the timing of apparent cranial fusion in *Triceratops* [7]. Several large specimens exhibit open sutures and unfused cranial elements (e.g., MOR 2702, MOR 2952, MOR 2971) and there are also smaller, less mature specimens in which several sutures of the skull appear closed (e.g., MOR 1120, MOR 2982, YPM 1822).Therefore, apparent cranial fusion is likely one of the least reliable ontogenetic indicators in *Triceratops*. It is also worth noting that YPM 1831 is, as O.C. Marsh [47] described it, “gigantic.” For these reasons, it is unlikely that YPM 1831 represents a subadult ‘*Torosaurus*.’

### Transitional Morphologies and Dinosaur Systematics

The ongoing dialogue regarding the taxonomic status of USNM 2412 highlights questions of whether suggested autapomorphies for some dinosaur taxa may in fact represent ontogenetic or other intraspecific variation. The potential for dinosaurs of different ontogenetic stages to be mistaken for distinct taxa has been recognized for over half a century [20]. In a landmark study, Dodson [21] demonstrated that lambeosaurines underwent dramatic changes in cranial morphology relatively late in ontogeny. This late stage morphological change was comparable to what is seen in some extant avian dinosaurs (birds) which retain an immature cranial morphology until late in development [21]. As such, specimens of ‘adult-sized’ dinosaurs may have still undergone considerable changes in morphology had they survived to reach full maturity (eg., [7], [10], [22]).

Ontogenetically transitional specimens can greatly affect interpretations of dinosaur systematics (e.g., [21], [22], [48], [49]). The largest, and presumably most mature, dinosaurs are relatively rare in the fossil record and when they are recovered are easily mistaken for new, distinct taxa based on the state of features which transform throughout development [23]. At the same time, specimens initially described as adults of new, small taxa [50], [51] may actually represent juveniles of other previously described taxa [48], [49]. In the absence of large sample sizes or monospecific bone beds, transitional specimens may be extremely difficult to recognize for what they are. The resulting overestimation of dinosaur diversity may produce an erroneous view of the paleoecology of these animals [21].

New insights into *Triceratops* ontogeny are the result of a very large sample size produced in part by extensive field exploration of the uppermost Cretaceous Hell Creek Formation [7], [23]. Each specimen of *Triceratops* is valuable for insights that may be provided regarding individual, ontogenetic, and stratigraphic variation [7]. The potential for transitional morphologies to be mistaken for unique taxa underscores the need for large scale field explorations in which numerous specimens of various growth stages are collected in order to test systematic hypotheses and clarify which morphological characters are in fact taxonomically informative.

### Conclusions

The evidence thus far collected supports the hypothesis that *Triceratops*, ‘*Torosaurus’*, and ‘*Nedoceratops’* are synonymous and that ‘*Torosaurus’* represents the mature morphology of *Triceratops*. Features suggested to indicate an ‘old adult’ ontogenetic status for USNM 2412 (rugose surface texture on frill, apparent fusion of cranial elements) are found in non-fully mature *Triceratops* and hence do not indicate that the ontogenetic transformation of USNM 2412 was complete. If anything, the small parietal fenestra found in this specimen supports the hypothesis that it was in the process of transitioning between the classic *Triceratops* and ‘*Torosaurus*’ morphologies. Farke [17] states that the hypothesis that USNM 2412 represents a taxon distinct from *Triceratops* would be refuted by “the identification of undisputed specimens of *Triceratops* that . . . preserve a mélange of character-states that are intermediate between known *Triceratops* specimens and *Nedoceratops* (pg. 6).” As we have presented here, there are numerous specimens of *Triceratops* which preserve such character-states in their nasal horn, squamosal, postorbital horn core and frill morphology thus refuting a distinct taxonomic position for ‘*Nedoceratops.*’

It might be argued that although the individual characters supposedly diagnostic of ‘*Nedoceratops*’ may be found to some degree in specimens of *Triceratops* and ‘*Torosaurus*’, it is the unique combination of these characters which distinguishes ‘*Nedoceratops*’ as a distinct taxon. But if this is true then nearly every specimen of *Triceratops* is referable to a distinct species and/or genus, for every specimen possesses some unique (if subtle) combination of characters which distinguishes it from others of the same taxon. This is the approach employed by Marsh and others which resulted in the naming of 16 species of *Triceratops* [2], [6].

Consideration of the radical nature of ontogenetic change that occurred in marginocephalians [7], [8], [22], [33], [34] is critical to systematic interpretations. Dinosaur specimens representing various transitional growth stages may easily be misinterpreted as distinct taxa [20], [21]. The debate which has surrounded USNM 2412 over the last century is inherently tied to the fact that it exhibits many intermediate features, and most likely represents an ontogenetically transitional morphology. Its small parietal fenestra is exactly what is predicted to be present at some point in *Triceratops* ontogeny as the fenestrae expanded and developed into the ‘*Torosaurus*’ morphology. Even if ‘*Torosaurus*’ was not synonymous with *Triceratops*, it would be more parsimonious to ascribe USNM 2412 to an immature ‘*Torosaurus*’ than to designate it as the holotype of a separate genus.

The synonymy of *Triceratops*, ‘*Torosaurus*’, and ‘*Nedoceratops*’ reduces perceived latest Cretaceous ceratopsid diversity, and affects our interpretations of these animals' paleoecology. This, along with other ontogenetic synonymies [22], [48], also supports the hypothesis that latest Maastrichtian dinosaur diversity was reduced relative to that found earlier in the Cretaceous [52], [53].

## Materials and Methods

The holotype skull of ‘*Nedoceratops hatcheri*’, USNM 2412, was examined first-hand in order to compare proposed autapomorphies with the condition expressed in other ceratopsids. Histological samples were prepared as has been previously described [7]. Specimens were embedded in polyester resin, sectioned with a precision saw, ground to a desired optical contrast using a lap wheel, and polished.

## References

[1] Marsh OC (1889). Notice of gigantic horned Dinosauria from the Cretaceous.. Am J Sci, series.

[2] Ostrom JH, Wellnhofer P (1986). The Munich specimen of *Triceratops* with a revision of the genus.. Zitteliana.

[3] Dodson P (1996). The Horned Dinosaurs.

[4] Forster CA (1996). Species resolution in *Triceratops*: cladistic and morphometric approaches.. J Vertebr Paleontol.

[5] Marsh OC (1891). Notice of new vertebrate fossils.. Am JSci, series.

[6] Ostrom JH, Wellnhofer P, Carpenter K, Currie PJ (1990). *Triceratops*: an example of flawed systematics;. Dinosaur Systematics: Approaches and Perspectives.

[7] Scannella JB, Horner JH (2010). *Torosaurus* Marsh, 1891, is *Triceratops* Marsh, 1889 (Ceratopsidae: Chasmosaurinae): synonymy through ontogeny.. J Vertebr Paleontol.

[8] Horner JR, Goodwin MB (2006). Major cranial changes during *Triceratops* ontogeny.. Proc R Society B.

[9] Horner JR, Goodwin MB (2008). Ontogeny of cranial epi-ossifications in *Triceratops*.. J Vertebr Paleontol.

[10] Horner JR, Lamm ET (2011). Ontogeny of the parietal frill of *Triceratops*: a preliminary histological analysis.. C R Pelevol.

[11] Lull RS (1905). Restoration of the horned dinosaur *Diceratops*.. Am J Sci.

[12] Lull RS (1933). A revision of the Ceratopsia or horned dinosaurs.. Yale Peabody Museum Memoir.

[13] Lehman TM (1998). A gigantic skull and skeleton of the horned dinosaur *Pentaceratops sternbergi* from New Mexico.. J Paleontol.

[14] Mateus O (2008). Two ornithischian dinosaurs renamed: *Microceratops* Bohlin, 1953 and *Diceratops* Lull, 1905.. J Paleontol.

[15] Ukrainsky AS (2007). A new replacement name for *Diceratops* Lull, 1905 (Reptilia: Ornithischia: Ceratopsidae).. Zoosystematica Rossica.

[16] Ukrainsky AS (2009). Synonymy of the genera *Nedoceratops* Ukrainsky, 2007 and *Diceratus* Mateus, 2008 (Reptilia: Ornithischia: Ceratopsidae).. Paleontologicheskii Zhurnal.

[17] Farke AA (2011). Anatomy and taxonomic status of the chasmosaurine ceratopsid *Nedoceratops hatcheri* from the Upper Cretaceous Lance Formation of Wyoming, U.S.A.. PLos One.

[18] Dodson P, Carpenter K, Currie PJ (1990). On the status of the ceratopsids *Monoclonius* and *Centrosaurus*.. Dinosaur systematics: approaches and perspectives.

[19] Sampson SD, Ryan MJ, Tanke DH (1997). Craniofacial ontogeny in centrosaurine dinosaurs (Ornithischia: Ceratopsidae): taxonomic and behavioral implications.. Zool J Linn Soc-Lond.

[20] Rozhdestvensky AK (1965). Growth changes in Asian dinosaurs and some problems of their taxonomy.. Palaeontol Zh.

[21] Dodson P (1975). Taxonomic implications of relative growth in lambeosaurine hadrosaurs.. Syst Zool.

[22] Horner JR, Goodwin MB (2009). Extreme cranial ontogeny in the Upper Cretaceous dinosaur *Pachycephalosaurus*.. PLoS ONE.

[23] Horner JR, Goodwin MB, Myrhvold N (2011). Dinosaur census reveals abundant *Tyrannosaurus* and rare ontogenetic stages in the Upper Cretaceous Hell Creek Formation (Maastrichtian), Montana, USA.. PLoSOne.

[24] Lehman TM, Carpenter K, Currie PJ (1990). The ceratopsian subfamily chasmosaurinae: sexual dimorphism and systematics.. Dinosaur systematics: approaches and perspectives.

[25] Dodson P, Currie PJ (1988). The smallest ceratopsid skull-Judith River Formation of Alberta.. Can J Earth Sci.

[26] Brown CM, Russell AP, Ryan MJ (2009). Pattern and transition of surficial bone texture of the centrosaurine frill and their ontogenetic and taxonomic implications.. J Vertebr Paleontol.

[27] Tanke DH, Farke AA, Carpenter K (2007). Bone resorption, bone lesions and extra cranial fenestrae in ceratopsid dinosaurs: a preliminary assessment.. Horns and beaks: ceratopsian and ornithopod dinosaurs.

[28] Farke AA, Carpenter K (2007). Cranial osteology and phylogenetic relationships of the chasmosaurine ceratopsid *Torosaurus latus*.. Horns and beaks: ceratopsian and ornithopod dinosaurs.

[29] Marsh OC (1898). New species of Ceratopsia.. Am J Sci, series 4.

[30] Forster CA (1993). Taxonomic validity of the ceratopsid dinosaur *Ugrosaurus olsoni* (Cobabe and Fastovsky).. J Paleontol.

[31] Cobabe EA, Fastovsky DE (1987). *Ugrosaurus olsoni*, a new ceratopsian (Reptilia: Ornithischia) from the Hell Creek Formation of eastern Montana.. J Paleontol.

[32] Wu X-C, Brinkman DB, Eberth DA, Braman DR (2007). A new ceratopsid dinosaur (Ornithischia) from the uppermost Horseshoe Canyon Formation (upper Maastrichtian), Alberta, Canada.. Can J Earth Sci.

[33] Sampson SD (1995). Two new horned dinosaurs from the Upper Cretaceous Two Medicine Formation of Montana; with a phylogenetic analysis of Centrosaurinae (Ornithischia: Ceratopsidae).. J Vertebr Paleontol.

[34] Currie PJ, Langston W, Tanke DH, Currie PJ, Langston W, Tanke DH (2008). A new species of *Pachyrhinosaurus* (Dinosauria, Ceratopsidae) from the Upper Cretaceous of Alberta, Canada.. A New Horned Dinosaur from an Upper Cretaceous Bone Bed in Alberta.

[35] Forster CA (1996). New information on the skull of *Triceratops*.. J Vertebr Paleontol.

[36] Forster CA, Sereno PC, Evans TW, Rowe T (1993). A complete skull of *Chasmosaurus mariscalensis* (Dinosauria: Ceratopsidae) from the Aguja Formation (Late Campanian) of West Texas.. J Vertebr Paleontol.

[37] Godfrey SJ, Holmes R (1995). Cranial morphology and systematics of *Chasmosaurus* (Dinosauria: Ceratopsidae) from the Upper Cretaceous of Western Canada.. J Vertebr Paleontol.

[38] Scannella JB, Fowler DW (2009). Anagenesis in *Triceratops*: evidence from a newly resolved stratigraphic framework for the Hell Creek Formation.. Cincinnati Museum Center Scientific Contributions.

[39] Johnson RE, Ostrom JH, Thomasson J (1995). The forelimb of *Torosaurus* and an analysis of the posture and gait of ceratopsians.. Functional Morphology in Vertebrate Paleontology.

[40] Scannella JB (2010). *Triceratops*: a model organism for deciphering dinosaur heterochrony.. J Vertebr Paleontol, SVP Program and Abstracts Book.

[41] Tsuihiji T (2010). Reconstructions of the axial muscle insertions in the occipital region of dinosaurs: evaluations of past hypotheses on marginocephalia and tyrannosauridae using the Extant Phylogenetic Bracket approach.. Ana Rec.

[42] Tumarkin-Deratzian AB, Ryan MJ, Chinnery-Allgeier BJ, Eberth DA (2010). Histological evaluation of ontogenetic bone surface texture changes in the frill of *Centrosaurus apertus*.. New perspectives on horned dinosaurs.

[43] Ryan MJ, Russell AP (2005). A new centrosaurine ceratopsid from the Oldman Formation of Alberta and its implications for centrosaurine taxonomy and systematic.. Can J Earth Sci.

[44] Francillon-Vieillot H, de Buffrénil V, Géraudie FJ, Meunier JY, Sire L, Carter JG (1990). Microstructure and mineralization of vertebrate skeletal tissues.. Skeletal Biomineralization: Patterns, Processes and Evolutionary Trends.

[45] Hieronymus TL (2006). Quantitative microanatomy of jaw muscle attachment in extant diapsids.. J Morphol.

[46] Goodwin MB, Horner JR, Ryan MJ, Chinnery-Allgeier BJ, Eberth DA (2010). Historical collecting bias and the fossil record of *Triceratops* in Montana.. New perspectives on horned dinosaurs.

[47] Marsh OC (1892). The skull of *Torosaurus.*. Am J Sci.

[48] Carr TD (1999). Craniofacial ontogeny in tyrannosauridae (Dinosauria, Coelurosauria).. J Vertebr Paleontol.

[49] Fowler DW, Woodward HN, Freedman EA, Larson PL, Horner JR (2011). Reanalysis of “*Raptorex kriegsteini*”: a juvenile tyrannosaurid dinosaur from Mongolia.. PLoS ONE.

[50] Bakker RT, Williams M, Currie PJ (1988). “*Nanotyrannus*, a new genus of pygmy tyrannosaur, from the latest Cretaceous of Montana.”. Hunteria.

[51] Sereno PC, Tan L, Brusatte SL, Kriegstein HJ, Zhao X (2009). Tyrannosaurid skeletal design first evolved at small body size.. Science.

[52] Archibald DJ (1996). Dinosaur extinction and the end of an era: what the fossils say.

[53] Campione NE, Evans DC (2011). Cranial growth and variation in Edmontosaurs (Dinosauria: Hadrosauridae): implications for Latest Cretaceous megaherbivore diversity in North America.. PLoS ONE.

